# Highly Permeable Fluorinated Polymer Nanocomposites for Plasmonic Hydrogen Sensing

**DOI:** 10.1021/acsami.1c01968

**Published:** 2021-04-28

**Authors:** Ida Östergren, Amir Masoud Pourrahimi, Iwan Darmadi, Robson da Silva, Alicja Stolaś, Sarah Lerch, Barbara Berke, Manuel Guizar-Sicairos, Marianne Liebi, Giacomo Foli, Vincenzo Palermo, Matteo Minelli, Kasper Moth-Poulsen, Christoph Langhammer, Christian Müller

**Affiliations:** † Department of Chemistry and Chemical Engineering, Chalmers University of Technology, Göteborg 412 96, Sweden; ‡ Department of Physics, Chalmers University of Technology, Göteborg 412 96, Sweden; § Paul Scherrer Institut, Villigen PSI 5232, Switzerland; ∥ Institute of Organic Synthesis and Photoreactivity, National Research Council, Bologna 40129, Italy; ⊥ Department of Industrial and Materials Science, Chalmers University of Technology, Göteborg 412 96, Sweden; # Department of Civil, Chemical, Environmental and Materials Engineering, Alma Mater Studiorum—University of Bologna, Bologna 40131, Italy

**Keywords:** fluorinated polymer, palladium nanoparticle, melt-processed nanocomposite, hydrogen permeability and diffusion, plasmonic sensing

## Abstract

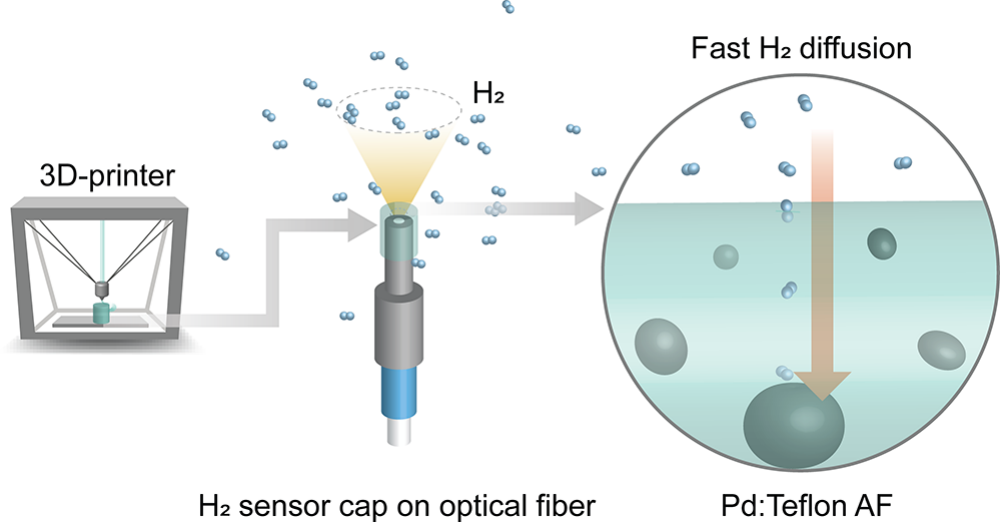

Hydrogen (H_2_) sensors that can be produced *en masse* with cost-effective manufacturing tools are critical for enabling safety in the emerging hydrogen economy. The use of melt-processed nanocomposites in this context would allow the combination of the advantages of plasmonic hydrogen detection with polymer technology; an approach which is held back by the slow diffusion of H_2_ through the polymer matrix. Here, we show that the use of an amorphous fluorinated polymer, compounded with colloidal Pd nanoparticles prepared by highly scalable continuous flow synthesis, results in nanocomposites that display a high H_2_ diffusion coefficient in the order of 10^–5^ cm^2^ s^–1^. As a result, plasmonic optical hydrogen detection with melt-pressed fluorinated polymer nanocomposites is no longer limited by the diffusion of the H_2_ analyte to the Pd nanoparticle transducer elements, despite a thickness of up to 100 μm, thereby enabling response times as short as 2.5 s at 100 mbar (≡10 vol. %) H_2_. Evidently, plasmonic sensors with a fast response time can be fabricated with thick, melt-processed nanocomposites, which paves the way for a new generation of robust H_2_ sensors.

## Introduction

Hydrogen gas (H_2_) is an efficient carbon-free energy carrier that can be extracted from sources that include natural gas,^1^ biomass,^2^ or water/wastewater.^3,4^ In contrast to the combustion of fossil fuels, the conversion of H_2_ to electrical energy in a fuel cell produces only water, without any CO_2_ emissions.^5^ Therefore, a transition from carbon-based fuels to H_2_ could ultimately play a key role in mitigating climate change.^6^ However, H_2_ gas is odorless, colorless, and highly flammable under ambient conditions when mixed with air, which causes considerable safety concerns. The ability to rapidly detect even minute amounts of H_2_ gas is therefore of paramount importance at all stages of the hydrogen energy cycle from production to transport, storage, and consumption.^7^ As a result, novel sensor technologies that can be used for on-the-spot detection of H_2_ leaks are currently in high demand.

A H_2_ sensor must be able to detect any leaks within a few seconds, depending on the specific application.^8^ Among different types of detection principles, optical sensing based on localized surface plasmon resonance (LSPR) is particularly promising because it offers a unique combination of high selectivity and accuracy, fast response, and spark-free operation due to remote readout.^8^ In this context, Pd is the most commonly used element and model system because it readily sorbs hydrogen without a sizable energy barrier and undergoes a reversible phase transformation from metal to metal hydride at room temperature, which gives rise to a sizable optical contrast.^9^ State-of-the-art H_2_ plasmonic sensors therefore comprise either pure Pd or Pd alloy nanoparticles^10^ that are shaped directly on a surface using nanolithography,^8,11^ are grown^12,13^ or deposited onto a surface using vacuum deposition,^14,15^ or are prepared by colloidal synthesis.^16^ Despite considerable advances in terms of both sensor fabrication and performance, the large-scale implementation of H_2_ plasmonic sensors is still lacking, partly due to cost and scalability issues with the nanofabrication methods used to produce two-dimensional (2D) arrays of hydrogen-sensitive plasmonic nanoparticles on flat surfaces. As a result, the attention is increasingly shifting to more cost-effective optical sensors that can be realized with scalable production methods.^17,18^ Therefore, we argue that it would be particularly desirable to harness the wealth of readily available polymer nanocomposite processing methods, such as solution processing,^19,20^ sputter deposition,^21−23^ and especially bulk processing methods, such as melt compounding.^16,24^

To this end, we have recently shown that plasmonic H_2_ sensors can be fabricated with three-dimensional (3D) geometries.^16^ Specifically, we prepared polymer nanocomposites composed of Pd nanoparticles, initially obtained by colloidal synthesis, and poly(methyl methacrylate) (PMMA), which we were able to shape using polymer processing methods such as extrusion, molding, and fused filament fabrication (FFF), a common 3D printing technique. While these Pd:PMMA nanocomposites could be used to fabricate fully functional H_2_ sensors with excellent long-term stability, the sensor elements suffered from a slow response and recovery time because of the slow H_2_ gas diffusion through the polymer matrix.^16^

Here, we demonstrate that the judicious selection of a polymer with a high permeability for H_2_ enables a fast response time as short as 2.5 s when exposing 100 μm-thick samples to a 100 mbar H_2_ pressure step from vacuum. This remarkable performance was enabled by the use of the amorphous fluorinated polymer poly(4,5-difluoro-2,2-bis(trifluoromethyl)-1,3-dioxole-*co*-tetrafluoroethylene) (Teflon AF) as the matrix material, which displays a substantially higher H_2_ permeability due to a considerably larger fractional free volume.^25,26^ In addition, the sensor shows astonishing sensitivity with an extremely low limit of detection (LOD) of 0.03 mbar (≡0.003 vol. % ≡ 30 ppm), which is among the best optical hydrogen sensors reported.^10^ We compounded Teflon AF with colloidal Pd nanoparticles, produced by a scalable continuous flow synthesis process,^27^ to create a highly hydrogen-permeable and hydrogen-sensitive nanocomposite material. Finally, we 3D-printed a fully functional sensor cap, which could be placed on a fiber optic connector and facilitated stable hydrogen sensing. The sensor exhibits outstanding stability under the 100 cycle (equivalent to 18 h) sensing test in a synthetic air background.

## Results and Discussion

**Figure 1:**
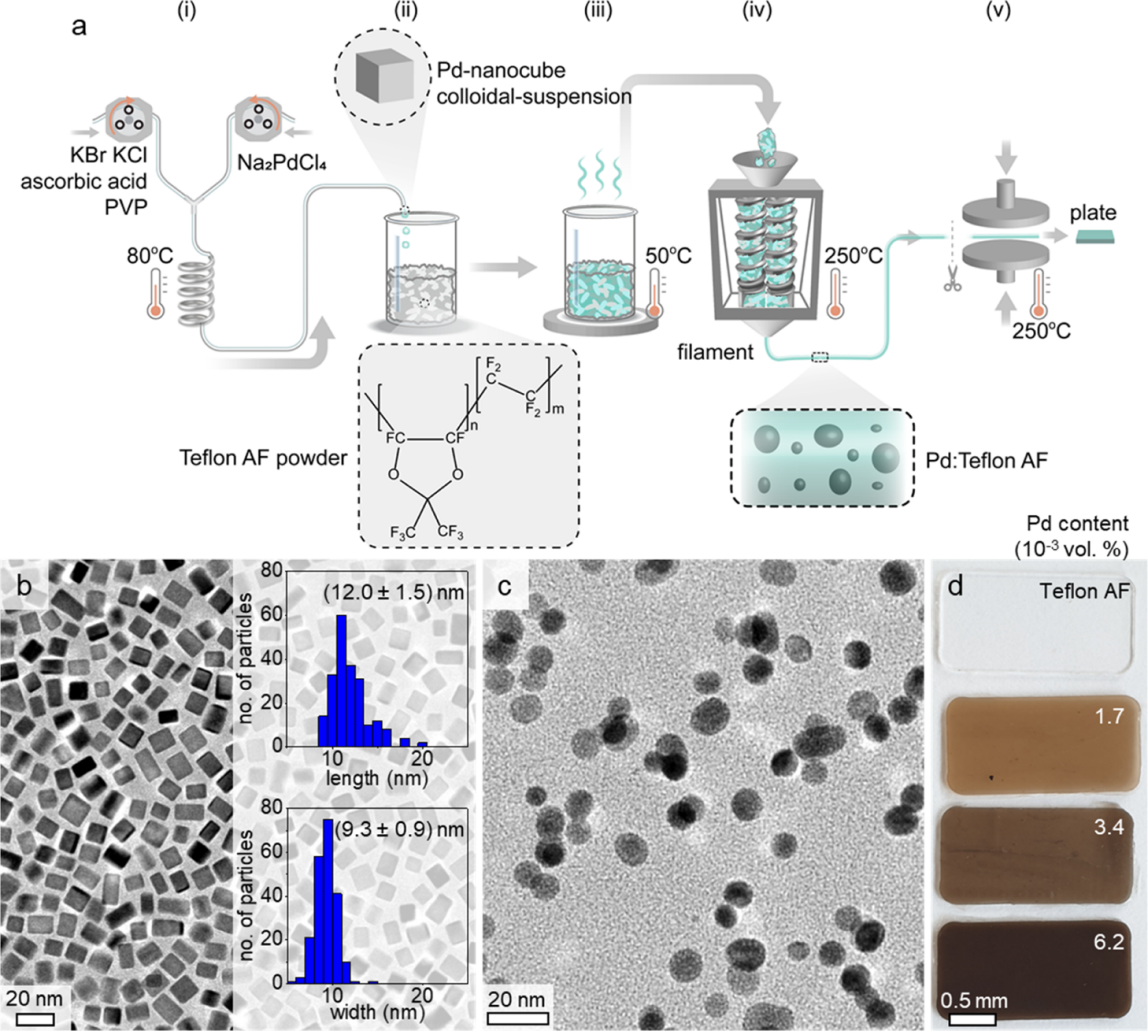
Polymer nanocomposite preparation. (a) Material preparation work flow entailing (i) flow synthesis of PVP-capped Pd nanocubes, (ii) mixing of the Pd nanocube suspension (isopropanol medium) with the polymer, Teflon AF, (iii) drying of the Pd nanoparticle:polymer mixture, (iv) melt extrusion, and (v) melt pressing; (b) TEM image of Pd nanocubes with size (rectangle with length and width) histograms; (c) TEM image of Pd nanoparticles in Teflon AF; and (d) melt-pressed 0.5 mm-thick plates of neat Teflon AF and its nanocomposites.

To prepare well-defined Pd nanoparticles in terms of size and shape, we used a continuous flow synthesis method,^27^ which provided single-crystal Pd nanoparticles coated/stabilized with poly(vinylpyrrolidinone) (PVP) (Figure 1a). Transmission electron microscopy (TEM) revealed that the obtained Pd single-crystal nanoparticles (enclosed by low-index^28^ facets with an edge truncation of 15%; Figure S1) had a uniform cubic shape with a narrow size distribution (Figure 1b). With a zeta potential of −35 mV at pH 5.6, the Pd nanocubes displayed a high degree of colloidal stability in the water-based reaction medium. To improve the compatibility with hydrophobic polymers such as Teflon AF targeted here, we carried out medium exchange by precipitation of the as-obtained Pd nanocubes from aqueous suspension with acetone, followed by redispersion in isopropanol.

The nanocomposite material was prepared by mixing the suspension of the Pd nanocubes with polymer powder, followed by evaporation of isopropanol at 40 to 50 °C overnight, resulting in polymer powder slurry with well-distributed Pd nanocubes. Finally, the material was molten in a microcompounder and extruded into filaments that could be used for melt pressing of thin plates, ranging from 50 to 650 μm in thickness (Figure 1a), or FFF-type 3D printing of more complex objects (will be discussed later). TEM images of cryo-fractured melt-pressed plates confirmed the presence of solitary Pd nanoparticles without aggregation (Figures 1c and S2). We note, however, that the Pd nanoparticles now had a more spherical shape, as compared to the nanocubes with sharp edges obtained from the synthesis (cf. Figure 1b). This change in shape was observed across entire samples (see Figure S2 for additional images). We attribute the restructuring of nanoparticles to the relatively high processing temperature of 250 °C (see Figure 1a), similar to the reconstruction of bare Pd nanoparticles at 150 to 220 °C reported by Pekkari et al.^27^ and not observed in our previous paper where the nanocomposite was processed at a lower temperature of 200 °C.^16^ The Pd content in the melt-pressed plates scaled with the amount of Pd present during the initial synthesis step (Figure S3a), which indicates that no material is lost during the various processing steps. Accordingly, the appearance of melt-pressed plates ranged from completely transparent in the case of neat Teflon AF to dark brown in the case of a nanocomposite containing 6.2 × 10^–3^ vol. % Pd (Figure 1d), due to the LSPR of the Pd nanoparticles.^29^ The LSPR frequency is independent of the Pd concentration (Figure S3b). The difference in appearance is due to higher light absorption, which increases with nanoparticle concentration (Beer–Lambert law^30,31^).

**Figure 2:**
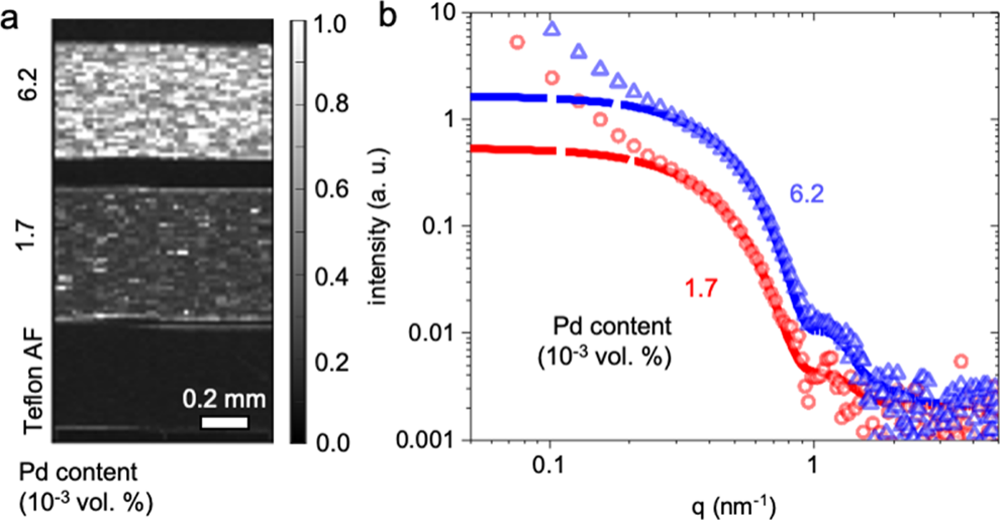
SAXS of Pd:Teflon AF nanocomposites. (a) Scanning-SAXS images of melt-pressed and (b) background-corrected SAXS scattering curves of Pd:Teflon AF nanocomposites (open symbols) with the corresponding ellipsoid fit (solid line); the scattering intensity of neat Teflon AF has been subtracted. The deviation from the fitted curve in the low *q* area is caused by the interactions between the nanoparticles and the Teflon AF matrix.

To assess the degree of dispersion of the Pd nanoparticles in melt-pressed Pd:Teflon AF nanocomposites at a more global scale, we carried out scanning small-angle X-ray scattering (SAXS) measurements (Figures 2 and S4). For this purpose, we prepared cross sections of melt-pressed plates containing 1.7 and 6.2 × 10^–3^ vol. % Pd. Using a 25 × 10 μm step size, we recorded scattering patterns over the chosen sample area of ∼0.5 × 1 mm^2^. The corresponding scanning-SAXS images (Figure 2a) show the average scattering intensity analyzed in a *q*-range of 0.2 to 0.69 nm^–1^. The scattering intensity appears similar at each scan position, which suggests that the Pd nanoparticles are homogeneously distributed in the Teflon AF matrix (Figure 2a; note that bright spots arise due to small local concentration differences). No preferential orientation was observed in the Pd:Teflon AF composites (Figure S4). Quantitative analysis of SAXS scattering curves revealed that the dispersed Pd nanoparticles had an average diameter between 8 and 11 nm obtained from ellipsoid fitting (Figure 2b), which is in agreement with our TEM analysis (cf. Figure 1c) and confirms that the majority of nanoparticles have undergone restructuring into ellipsoids and are well dispersed, that is, no or only very few aggregates are present in the nanocomposite.

**Figure 3:**
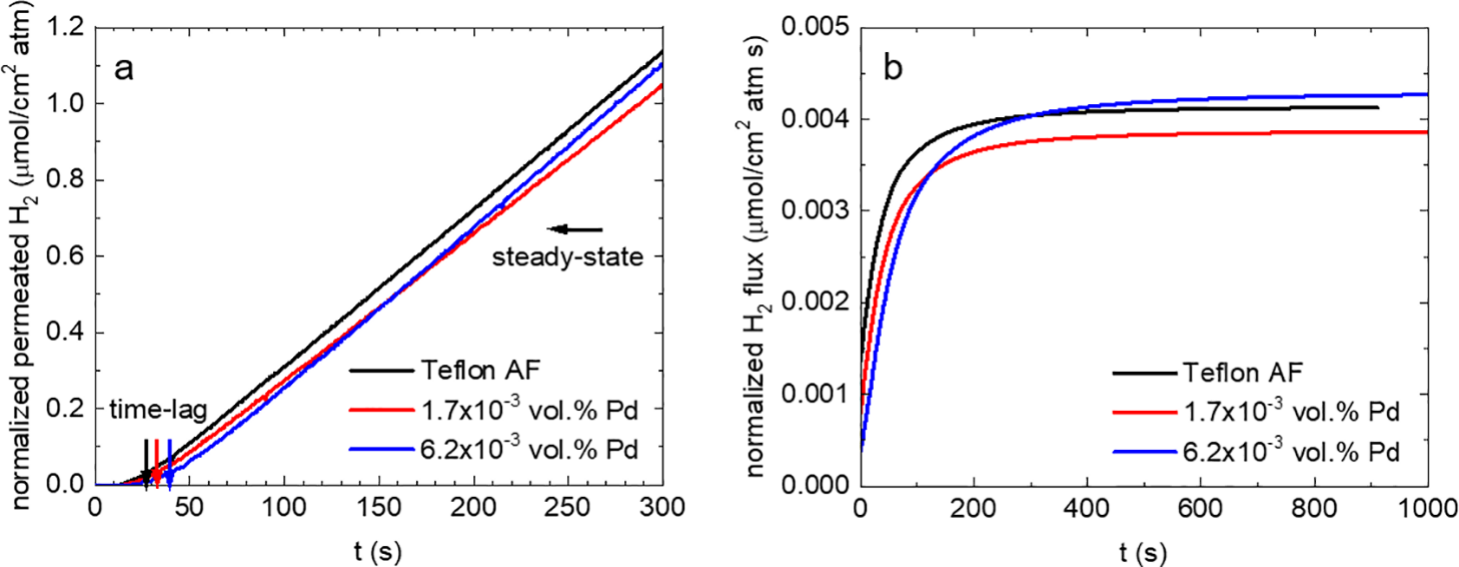
H_2_ permeability measurement based on the time-lag method. (a) Normalized permeated H_2_ and (b) normalized flux of H_2_ per unit area through 600 μm ± 25 μm-thick melt-pressed plates of neat Teflon AF and Pd:Teflon AF nanocomposites (the flux was normalized with respect to the sample thickness).

To determine the permeability of H_2_ through melt-pressed plates of neat Teflon AF and of nanocomposites containing the Pd nanoparticles, we used a custom-made setup that allows measuring of the time lag of gas transport through polymer films, which separate the feed and the initially empty permeate compartment.^32^ The feed side of the sample is exposed to H_2_, which absorbs and diffuses through the plate and then desorbs on the permeate side, leading to a gradual increase in pressure until the steady-state conditions are obtained (linear increase with time of permeated H_2_ molecules, i.e., constant flux). The time lag at the beginning of the measurement and the slope in the steady-state region allow us to determine the diffusion coefficient *D* and the permeability *P*, respectively (Figure 3). To benchmark the high permeability of Teflon AF, we also included semicrystalline poly(vinylidene fluoride) (PVDF) and amorphous PMMA in our study. For neat Teflon AF, we obtain *D* ≈ 2.3 × 10^–5^ cm^2^ s^–1^ and *P* ≈ 745 barrer, which are both considerably higher than values obtained for PMMA or PVDF (Table 1), indicating that Teflon AF is a superior choice for the realization of nanocomposites that are to be used for rapid H_2_ sensing. We observe that the presence of Pd nanoparticles in the Teflon AF matrix does not affect *P* and only slightly reduces *D* (Table 1), which is in agreement with studies of Pd in other polymers.^19^ The higher diffusion coefficient of Teflon AF compared to PMMA, which are both amorphous [cf. differential scanning calorimetry (DSC) thermograms in Figure S5] can be rationalized by considering the fractional free volume (FFV) since *D* ∝ e^–*B*/FFV^,^28^ where *B* is a constant. Teflon AF tends to pack poorly in the glassy state,^26,33^ which gives rise to a higher FFV and hence *D* than PMMA. Instead, the lower diffusion coefficient of PVDF can be ascribed to the semicrystalline nature of the polymer (Figure S5).

**Table 1:** H_2_ Permeability *P* and Diffusivity *D* from the Time-Lag Method, Calculated after a Long Desorption Time (Overnight) and Characteristic Diffusion Time CDT = *d*^2^/6*D* of Plates with Thickness *d*

material	***d*** (μm)	***P*** (barrer)	***D*** (cm^2^/s)	**CDT** (s)
PMMA	262 ± 9	4.2 ± 0.2	(6.6 ± 0.4) ×10^–7^	177 ± 24
Teflon AF	589 ± 16	745 ± 2	(2.32 ± 0.04) ×10^–5^	25 ± 1
+1.7 × 10^–3^ vol. % Pd^a^	586 ± 9	706 ± 2	(1.9 ± 0.1) ×10^–5^	30 ± 1
+6.2 × 10^–3^ vol. % Pd^a^	608 ± 16	799 ± 5	(1.5 ± 0.2) ×10^–5^	41 ± 4
PVDF	250 ± 38	0.48 ± 0.02	(5.9 ± 0.2) ×10^–8^	1780 ± 55

a For Teflon AF nanocomposites, *D* represents an effective value that also accounts for the hydride formation reaction. 1 barrer = 10^-10^ cm^3^ (STP)cm/(cm^2^s cmHg).

**Figure 4:**
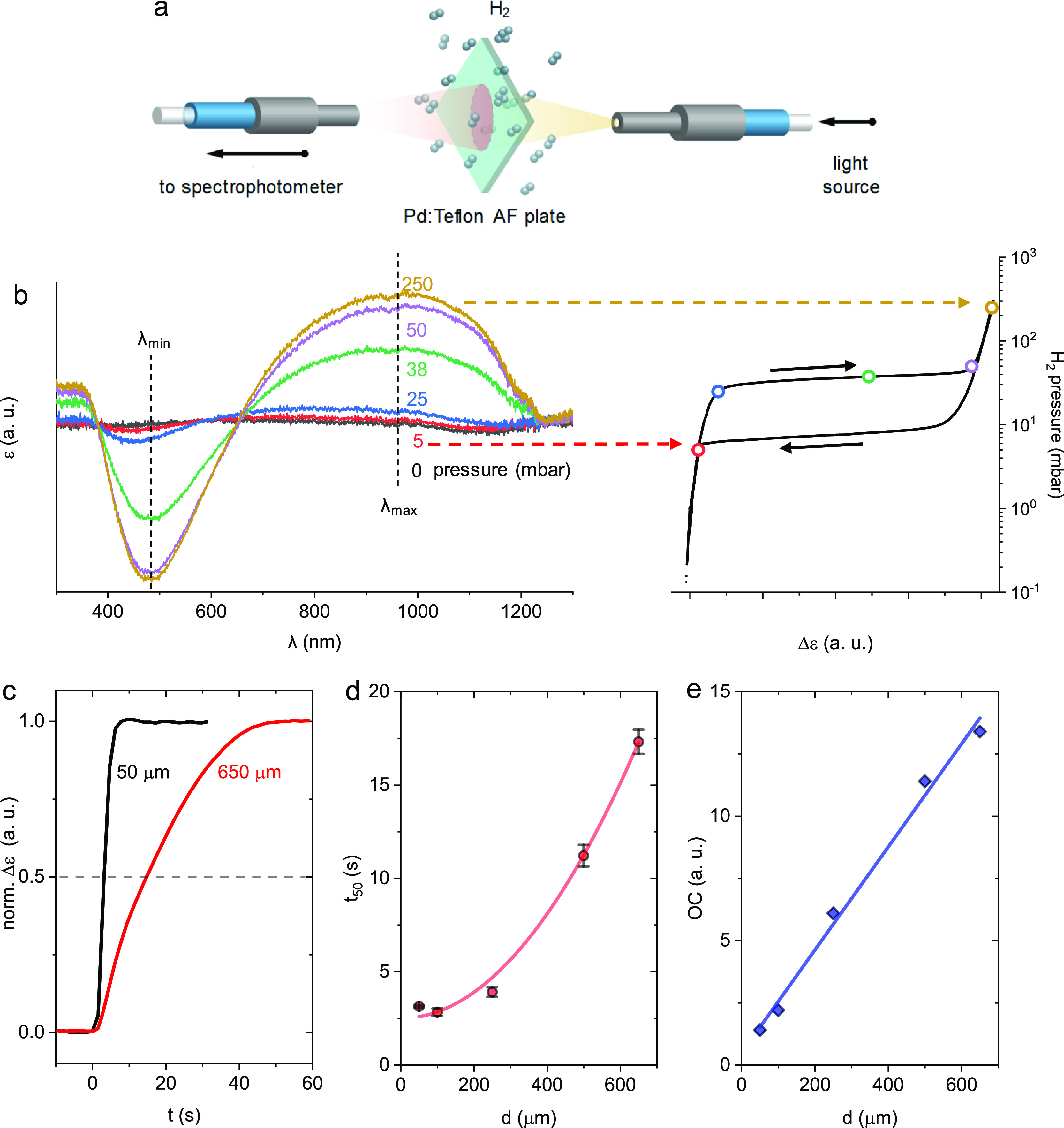
Plasmonic H_2_ sensing. (a) Schematic of optical H_2_ detection via extinction measurements through a melt-pressed nanocomposite plate; (b) selected self-referenced extinction spectra of a 100 μm-thick melt-pressed plate composed of Pd:Teflon AF (12.5 × 10^–3^ vol. % Pd) during exposure to increasing 0–250 mbar H_2_ pressure at 30 °C together with their pressure–composition isotherm constructed using the difference in extinction Δε = ε(λ_max_) – ε(λ_min_) as the readout descriptor. A similar isotherm construction is also shown for decreasing hydrogen pressure from 250–0 mbar H_2_. Here, self-referenced extinction corresponds to the use of an extinction spectrum measured from a plate in the nonhydrogenated state as the optical reference. (c) Normalized Δε of 50 and 650 μm-thick melt-pressed plates of Pd:Teflon AF (3.4 × 10^–3^ vol. % Pd) upon a stepwise increase in H_2_ pressure from 0 to 100 mbar H_2_ (the H_2_ valve opens at *t* = 0); (d) H_2_ absorption time or “response time” of the plate, *t*_50_, defined as the time it takes to reach 50% of the total signal change to the new steady state versus the plate thickness *d* (error bars are the standard deviation of five identical measurements). Also plotted is a quadratic fit *t*_50_ = 3.5 × 10^–5^ s μm^–2^*d*^2^ + 2.5 s (red line) and (e) optical contrast (OC) (see the main text for definition) as a function of plate thickness *d*, plotted together with a linear fit OC = 0.02 μm^–1^*d* + 0.48 (blue line).

As the next step, we evaluated the effectiveness of Pd:Teflon AF nanocomposites for plasmonic optical H_2_ sensing by spectrally resolved monitoring of the optical extinction of a melt-pressed plate during H_2_ exposure at different pressures (Figure 4a). The spectra feature distinct changes in the self-referenced extinction, ε, upon exposure to H_2_, with a minimum and maximum emerging at λ_min_ ≈ 480 nm and at λ_max_ ≈ 975 nm, respectively (Figure 4b). Here, self-referenced extinction means that the measured extinction signal is normalized with respect to the first spectrum measured for the composite in the nonhydrogenated state. We chose to use the difference in extinction Δε = ε(λ_max_) – ε(λ_min_) as the descriptor for the optical response to hydrogen to cancel the spectrum baseline drift. Using this descriptor, we then constructed a pressure-composition isotherm (Figure 4b), which reproduces the characteristics of the palladium–hydrogen system, with the solid solution α-phase present at low H_2_ pressure, the β-phase present at high H_2_ pressure, and an equilibrium plateau where both α- and β-phase coexist during the first-order phase transformation.^9^ We also note that hysteresis occurs during pressure cycling, showing that the hydride decomposition occurs at a lower pressure as compared to palladium-hydride formation, as a consequence of lattice strain and the corresponding energy barriers due to the presence of hydrogen in the host lattice.^9^ The characteristic shape of the isotherm also confirms that the observed changes in extinction upon H_2_ exposure arise due to absorption/desorption of hydrogen by the Pd nanoparticles, as anticipated. In addition, a polymer coating is known to shift the Pd plateau pressure due to induced strain.^8^ Therefore, the plateau pressure shown in Figure 4b is likely to be distinct from that of Pd without Teflon AF. Furthermore, the pressure–composition isotherms recorded for different Pd concentrations (Figure S6) do not indicate a correlation between the plateau pressures and the Pd concentration. We explain the variation of the plateau pressure with batch-to-batch differences of the nanoparticles, not only in shape and size but also in the internal nanostructure.^34,35^

Now turning our analysis and discussion to the targeted application of the nanocomposite as optical plasmonic hydrogen sensors, we focus on the assessment of the response and recovery times, since they are vital aspects of a H_2_ sensor. Therefore, we investigated the H_2_ absorption and desorption kinetics of 50 to 650 μm-thick plates, by monitoring Δε upon a stepwise increase in H_2_ pressure from 0 to 100 mbar (Figures 4c and S7). We define the response time *t*_50_ as the time it takes to reach 50% of the total signal of the new steady state (cf. Figure 4c) and observe that *t*_50_ scales quadratically with the plate thickness, *d* (Figure 4d), indicating that the response is controlled by the diffusion of H_2_ through the polymer matrix. Applying our previously established model^16^ for the diffusion-limited H_2_ sorption kinetics of nanocomposites to this data set, we obtain a diffusion coefficient of *D* ≈ 1.6 × 10^–5^ cm^2^ s^–1^, which is in excellent agreement with values obtained from direct gas permeation measurements (Table 1). A similar analysis of the desorption kinetics likewise shows a quadratic dependence of *t*_50_ ∝ *d*^2^ and yields a comparable value of *D* ≈ 3.1 × 10^–5^ cm^2^ s^–1^ (Figure S8). We also studied the impact of Pd concentration on *t*_50_ using 100 μm-thick plates (Figure S9). Both absorption and desorption *t*_50_ do not depend on the concentration of Pd nanoparticles (Figure S10). This can be understood from two perspectives. First, the characteristic diffusion time (CDT = *d*^2^/6*D*) of hydrogen through a thickness of 100 μm is about 0.8 s (cf. Table 1), which is less than the typical H_2_ absorption time of a few seconds, reported for similar Pd nanoparticles; hence, the H_2_ concentration gradient through the plate is negligible.^16^ Second, the investigated Pd concentration range is much lower than the amount of the hydrogen dissolved in the polymer matrix. Consequently, these two factors cause the apparent hydrogenation time to only depend on the hydrogen uptake rate per nanoparticle.^16^

The optical contrast OC = Δε_PdHx_ – Δε_Pd_, which directly determines the sensitivity and signal-to-noise ratio of a sensor, is here defined as the difference in extinction Δε_PdHx_ when Pd is fully hydrogenated at a pressure of 100 mbar H_2_ minus the difference in extinction Δε_Pd_ of the nonhydrogenated state. We find that the optical contrast increases linearly with plate thickness at constant Pd concentration, that is, OC ∝ *d* (Figure 4e). A similar trend can also be achieved by increasing the Pd nanoparticle loading, in agreement with the Beer–Lambert law.^30,31^ These two strategies to enhance the optical contrast will be relevant for operation at elevated temperatures, for example, in connection to a fuel cell because the sensitivity of Pd-hydride based sensors tends to decrease due to lower hydrogen solubility.^36^

However, while a thick plate results in a high signal-to-noise ratio, it also increases the response time of the sensor due to longer diffusion paths. Therefore, we argue that a thickness of 100 μm is the optimal choice with a *t*_50_ ≈ 2.5 s (pressure step of 0 to 100 mbar), since it does not decrease considerably for thinner samples (cf. Figure 4d). The optical contrast can instead be adjusted by selecting a sufficiently high Pd nanoparticle concentration, which does not influence *t*_50_ (Figure S10). We note that the response time of the sensor with optimal thickness is higher compared to state-of-the-art hydrogen sensors.^10^ However, since in our case, the response time is limited by the hydrogen absorption kinetics of the Pd nanoparticles, in the next generation versions of sensors, it can be further improved by, for example, using a PdAu alloy,^37^ Pd nanoparticles with specific facets/vertices,^38^ or a nonhalide-based nanoparticle stabilizer.^39^

To examine the LOD of the sensor, we tested the response of 50 and 500 μm-thick samples to low hydrogen pressures in the α-phase regime of the Pd (Figure S11). We define the LOD as equal to three times the noise level (LOD = 3σ = 0.03 a.u.). The 50 and 500 μm samples feature LODs of 0.84 mbar (≡0.084 vol. % ≡ 840 ppm under ambient conditions) and 0.03 mbar (≡0.003 vol. % ≡ 30 ppm), respectively. To put these values into perspective, we note that both these LODs are below the limit required by some national agencies (0.1 vol. %).^10^ Furthermore, our results indicate that one strategy to amplify the optical response (i.e., the LOD) is to further increase the plate thickness.

Furthermore, it is worth discussing that the sensor sensitivity can be further improved by increasing the nanoparticle size. The nanoparticles we used in this study are very small and their LSPR wavelength is in the UV spectral range (Figure S3b). At the same time, the sensitivity of plasmonic Pd-based sensors is proportional to the LSPR wavelength of the particles in the nonhydrogenated state, which, in turn, is proportional to the nanoparticle size.^40,41^ In this study, we chose to work with Pd nanoparticles that are 10 nm in size because the synthesis time increases for larger nanoparticles.

**Figure 5:**
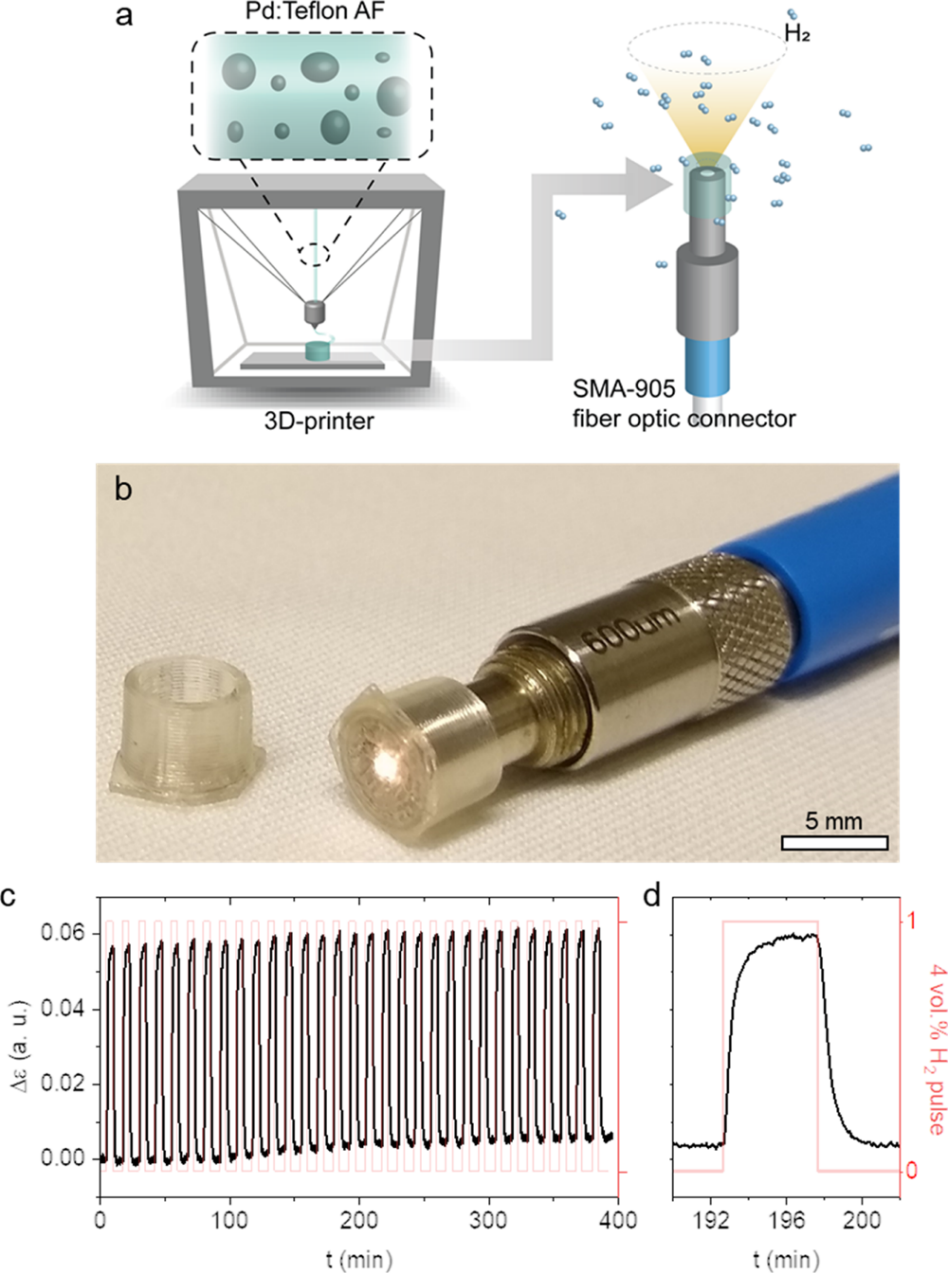
Hydrogen sensing with an optical fiber cap produced by FFF. (a) Fabrication scheme of sensor caps, which are designed to fit by “plug-and-play” onto standard SMA 905 fiber optic connectors; (b) photograph of a sensor cap on an SMA 905 connector, fabricated using a Pd:Teflon AF (3.4 × 10^–3^ vol. % Pd) filament (note that only the exposed part to hydrogen, i.e., the bottom part, is made of the Teflon nanocomposite); (c) sensor response, Δε, during cyclic exposure to 4 vol. % hydrogen in synthetic air; and (d) inset: 15th cycle.

In a last set of experiments, we used FFF to 3D-print sensor caps with a bottom wall thickness of 200 μm. The cap was fit by “plug and play” onto an SMA 905 fiber optic connector (Figure 5a,b), which was inserted into a custom-built test chamber (cf. ref (16) for setup and methodology) in which the sensor can be exposed to pulses of 4 vol. % H_2_ in synthetic air in a constant flow configuration to emulate a hydrogen leak. Gratifyingly, the sensor exhibits a stable and reversible response for the investigated cyclic exposure (Figures 5c,d and S12). The baseline drift, which appears in Figure 5c, is most likely induced by external factors, such as temperature fluctuation or instability of the light source during the test. The observed apparent longer response and recovery time compared to our previous tests, Figure 4, is the consequence of the gas exchange constant of the chamber, rather than reflecting the intrinsic response/recovery times of the sensor material. We also carried out experiments where we added 500 ppm CO or H_2_O vapor (75% relative humidity) to the synthetic air mixture and found that the optical response/recovery time of Pd:Teflon AF to H_2_ slowed down dramatically (not shown). CO and H_2_O molecules are known to strongly bind to Pd surfaces, thereby blocking H_2_ sorption. We explain the inability of Teflon AF to protect the Pd nanoparticles with the relatively large free volume of the polymer, which not only facilitates rapid diffusion of H_2_ but also allows larger species such as CO and H_2_O to traverse the matrix.

## Conclusions

We have prepared nanocomposites of Pd nanoparticles, produced by continuous flow synthesis, and the fluorinated amorphous polymer Teflon AF. Thanks to the high fractional free volume of Teflon AF, the diffusion of H_2_ through the polymer matrix is sufficiently rapid to not limit the response time of plasmonic sensing based on palladium hydride formation. Melt-pressed plates of Pd:Teflon AF nanocomposites with a thickness of 100 μm display a short response time of only *t*_50_ ≈ 2.5 s upon exposure to H_2_ gas (stepwise increase from 0 to 100 mbar in the vacuum background). The sensor exhibits a low LOD down to 30 ppm. Finally, we have manufactured sensor caps with FFF, a common 3D printing technique, which can be placed on a standard fiber optic connector and facilitate robust hydrogen sensing. A printed cap displays robust sensing in a long term test of 100 exposures (≡18 h) to 4 vol. % H_2_ in synthetic air. With this demonstration, we argue that melt-processing of Pd:polymer nanocomposites is a viable route toward the realization of plasmonic plastic H_2_ sensors.

## Experimental Section

### Synthesis of PVP-Capped Nanocubes Using Flow Chemistry

All chemicals used were of analytical grade (purity >99%) and used as received from Sigma-Aldrich. All solutions were prepared with MilliQ-water with a resistivity of 18.2 MΩ cm^–1^. The automated segmented flow synthesis was performed using a flow system featuring two peristaltic pumps, an air-heated reaction zone and full automation control using the connected computer with integrated software (Vapourtec E-series). Reagents were pumped in high-purity-grade perfluoroalkoxy (PFA) tubes (i.d. 1 mm). An aqueous solution of sodium chloropalladate (Na_2_PdCl_4_) (63.3 mM) was pumped with a peristaltic pump at a rate of 0.091 mL min^–1^ and was interfaced in a (ethylene tetrafluoroethene, o.d. 1.57 mm) T-junction with a mixture of potassium chloride 307.1 mM, potassium bromide 5.41 mM, polyvinylpyrrolidone 118.2 mM, and ascorbic acid 42.7 mM, pumped at a rate of 0.242 mL min^–1^. The outlet of the T-junction was connected to a coiled flow reactor (10 mL; high-purity-grade PFA tubes with i.d. 1 mm) which was kept at a constant temperature (80 °C) by hot air (Vapourtec system) and collected in a vial. For medium exchange from water to isopropanol, the Pd suspension was mixed with acetone in the ratio 3:1 (acetone/aqueous suspension of Pd nanocubes). For instance, 10 mL of aqueous suspension of Pd nanocubes was placed in a 50 mL centrifuge tube and 30 mL of acetone was added. The mixture was centrifuged at a speed of 3000 rpm for 5 min. The supernatant was discarded, and 10 mL of isopropanol was added. The suspension of nanoparticles in isopropanol was placed in an ultrasound bath for 5 min to improve the colloidal stability.

### Polymer Nanocomposite Fabrication (Melt Extrusion, Melt Pressing, and 3D Printing)

Poly[4,5-difluoro-2,2-bis(trifluoromethyl)-1,3-dioxole-*co*-tetrafluoroethylene] (AF 1600, here called Teflon AF) with a dioxole content of 65 mol % and a density of 1.78 g cm^–3^ was obtained from Chemours; PMMA with a density of 1.2 g cm^–3^, weight-average molecular weight *M*_w_ ≈ 75 kg mol^–1^, and a polydispersity index of 2.8 was obtained from Polysciences Inc.; and PVDF with a density of 1.78 g cm^–3^ and a melt flow index of 40 g/10 min (230 °C, 2.16 kg) was obtained from Solvay Solexis SAS (grade Solef 1006). Different volumes of Pd nanocubes dispersed in isopropanol (0.3–3 mL) were added to 2 cm^3^ polymer powder and kept at 50 °C overnight for drying (removal of isopropanol). The dry mixtures were compounded for 5 min in an Xplore Microcompounder MC5 at 250 °C. The extrudates were melt-pressed at the same temperatures and 100 kN for 5 min, resulting in a thickness ranging from 50 to 650 μm.

### Fused Filament Fabrication

3D printing was carried out with a Mass Portal Pharaoh XD printer using a nozzle temperature of 260 °C, a build-plate temperature of 60 °C, and a printing speed of 1000 mm min^–1^.

### Elemental Analysis

Elemental analysis was carried out at Mikrolab Kolbe, Germany.

### Transmission Electron Microscopy

TEM was carried out with a Tecnai T20 microscope with a LaB6 gun, operating at 200 kV, or a Titan 80-300 microscope with a field emission gun, operating at 300 kV. The Pd suspension was dropped on a pure carbon 200 mesh copper grid (Ted Pella), while melt-pressed Pd:Teflon AF nanocomposites were microtomed and placed on a Lacey 400 mesh copper grid.

### UV–Vis Absorption Spectroscopy

UV–vis absorbance spectra of melt-pressed plates were recorded with a Lambda 1050 spectrophotometer from PerkinElmer.

### Scanning SAXS

Scanning SAXS measurements were carried out at the cSAXS (X12SA) beamline of the Swiss Light Source at the Paul Scherrer Institute (PSI) in Villigen PSI, Switzerland. The samples were scanned by a beam with an energy of 11.2 keV, which was defined by a fixed-exit double-crystal Si(111) monochromator and focused to a 28 × 7.5 μm^2^ beam size. A 2 m-long flight tube under vacuum was inserted between the sample and the detector to minimize air scattering and X-ray absorption. The sample-to-detector distance was 2.19 m calibrated by silver behenate. A Pilatus 2M (1475 × 1679 pixels; pixel size: 172 × 172 μm^2^) detector was used to acquire 2D small-angle scattering patterns.^42^ Simultaneously, the transmitted beam was measured by the fluorescence signal from the steel beamstop. The sample slices (thickness of 200 μm) were placed on a polyimide (Kapton) film (Goodfellow Corp., Cambridge, UK). A raster scan was performed on a chosen sample area of 2 × 1 mm^2^ with a step size of 25 × 10 μm^2^ and an exposure time of 0.1 s. Data processing was carried out using the “cSAXS scanning SAXS package” developed by the CXS group, Paul Scherrer Institute, Switzerland.^43^ To determine the size of the Pd particles, form factor fitting was carried out using SASView 4.2.0 on 20 points for each sample. The signal of the Kapton foil and the corresponding pure (nanoparticle-free) polymer matrix was subtracted before the fitting procedure to remove the background signal. The ellipsoid model with the hard-sphere structure factor was chosen for the fitting. A polydispersity of 0.1, determined by trial and error, was applied for determining the size of the ellipsoid.

### Thermal Analysis

DSC was carried out with a DSC2 from Mettler Toledo under nitrogen (100 mL min^–1^), between 25 and 300 °C, at a scan rate of 10 °C min^–1^. The sample weight was 5–10 mg.

### Hydrogen Permeability

The hydrogen transport properties were determined by means of direct permeation experiments using a manometric technique (ASTM Standard norm D 1434), and the penetrant flux through the samples was obtained from the pressure increase in a calibrated closed volume, starting from high vacuum conditions.^44^ A capacitive gauge (Edwards Barocel, 0–100 mbar range, sensitivity 0.01 mbar) measured the gas pressure in the downstream chamber, while the upstream pressure was kept constant at approximately 1.2–1.5 bar (Druck, 0–10 bar). Tests were carried out under isothermal conditions at 30 °C in a thermostatic chamber (PID, ±0.1 K). Samples were first placed in the sample holder (Millipore, permeation area 2.2 cm^2^) set across the two closed volumes and treated overnight under vacuum to remove all possible adsorbed species. The tests were performed out by connecting a high-pressure chamber with the specimen and were stopped only after steady-state conditions are achieved, observed as a linear increase in downstream pressure over time. Each test was repeated at least twice to ensure the reproducibility of the data obtained. The gas permeability *P* was calculated as follows
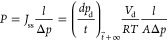
1where *J*_ss_ is the penetrant molar flux per unit area (at the steady state), Δ*p* is the pressure difference, *p*_d_ is the downstream pressure, *V*_d_ is the calibrated volume of the downstream side, and *A* is the membrane area. Diffusion kinetics were evaluated by the time-lag method, which represents the process characteristic time, and the value of θ_L_ was determined as the intercept on the time axis of the linear *p*_d_ versus *t* behavior at long times, once steady-state conditions were attained; the diffusivity *D* was thus calculated as^45^
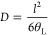
2

### Hydrogen Sensing Measurements

H_2_ sensing was carried out with two platforms: (i) a home-built vacuum chamber, for the experiment in Figure 4, and (ii) a mini flow reactor, for the experiment in Figure 5. The setups are described in detail elsewhere.^16^ Experiment (i) was carried out at 30 °C using a feedback-loop heating system comprising a heating coil, a power supply, and a PID thermo-controller (Eurotherm 3216). Experiment (ii) was performed at 21 °C (the ambient lab temperature). Both experiments were executed in the transmission mode, where a polychromatic halogen light (Avantes AvaLight-Hal Mini) was directed toward the sensor and the transmitted light was monitored using a visible light-range spectrophotometer (Avantes SensLine AvaSpec-2048XL). In experiment (i), the optical response of the sensor was monitored during the quasi-static, (de)increasing hydrogen pressure controlled using leak valves (for the isotherm measurement), and during a sudden hydrogen pressure change using pneumatic valves (for the kinetic measurement). In the case of experiment (ii), the optical response was monitored during cycles of exposure to 4 vol. % H_2_ (diluted in synthetic air and total flow = 200 ml min^–1^). The gas flow and mixture were controlled by mass flow controllers (Bronkhorst LowΔP).

## Supporting Information Available

TEM images of the Pd single crystal and Pd nanoparticles in Teflon AF, UV–vis absorbance spectra of Pd:Teflon AF plates; elemental analysis for obtaining the Pd concentration in Pd:Teflon AF plates; SAXS of Pd:Teflon AF nanocomposites; DSC melting thermograms for PMMA, Teflon AF, and PVDF; isotherms of different Pd loading Pd:Teflon plates; Δε, *t*_50_, and optical contrast of Pd:Teflon AF as functions of thickness and Pd content upon hydrogen absorption and desorption; Pd:Teflon sensor response at low hydrogen pressures and the limit of detection and *t*_50_ during hydrogen cycling exposure for the 3D-printed sensor (PDF)
