# Supplementary material for: Highly Permeable Fluorinated Polymer Nanocomposites for Plasmonic Hydrogen Sensing
Source: ACS Appl Mater Interfaces. 2021 Apr 28;13(18):21724–32. doi: 10.1021/acsami.1c01968 (PMC8289187; doi:10.1021/acsami.1c01968)
Supplement: Supplementary file 1 — am1c01968_si_001.pdf [file am1c01968_si_001.pdf]

Supporting Information

A Highly Permeable Fluorinated Polymer Nanocomposite for Plasmonic Hydrogen Sensing

I. Östergren,[1+] A. M. Pourrahimi,[1+] I. Darmadi,[2+] R. R. da Silva,[1] A. Stolaś,[1] S. Lerch,[1] B. Berke,[2] M. Guizar-Sicairos,[3] M. Liebi,[2] G. Foli,[4] V. Palermo,[4,5] M. Minelli,[6] K. Moth-Poulsen,[1*] C. Langhammer,[2*] C. Müller[1*]

[1]Department of Chemistry and Chemical Engineering, Chalmers University of Technology, 412 96 Göteborg, Sweden

[2]Department of Physics, Chalmers University of Technology, 412 96 Göteborg, Sweden

[3]Paul Scherrer Institut, PSI-Villigen, Switzerland

[4] Institute of Organic Synthesis and Photoreactivity, National Research Council, 40129, Bologna, Italy

[5] Department of Industrial and Materials Science, Chalmers University of Technology, 412 96 Göteborg, Sweden

[6]Department of Civil, Chemical, Environmental and Materials Engineering, Alma Mater Studiorum - University of Bologna, 40131 Bologna, Italy

[+]equal contribution

*Correspondence to: moth-poulsen@chalmers.se, christian.muller@chalmers.se, clangham@chalmers.se

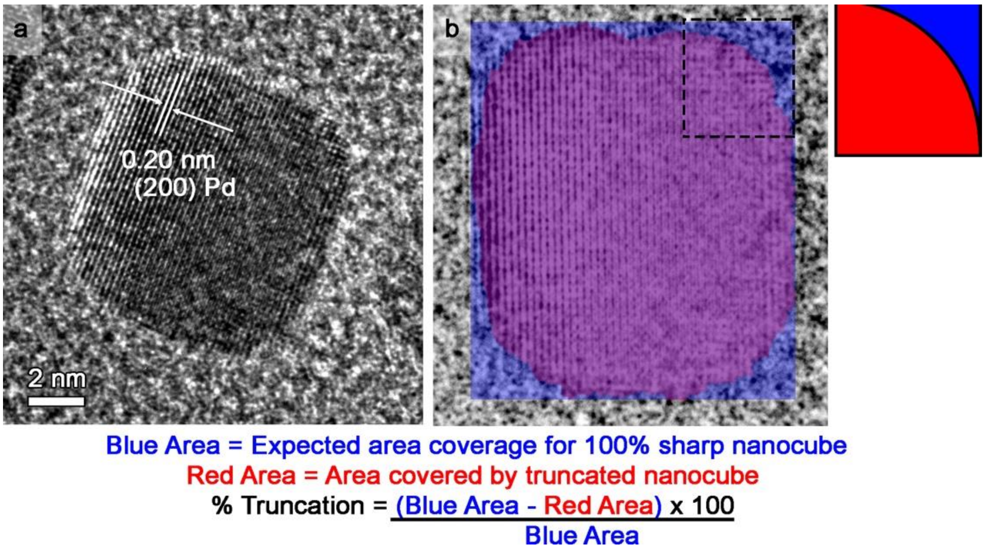

Figure S1. a) High-resolution TEM image of a single Pd nanocube, and b) calculation of edge-truncation degree of a single nanocube. The nanocubes showed ~15% of edge-truncation.

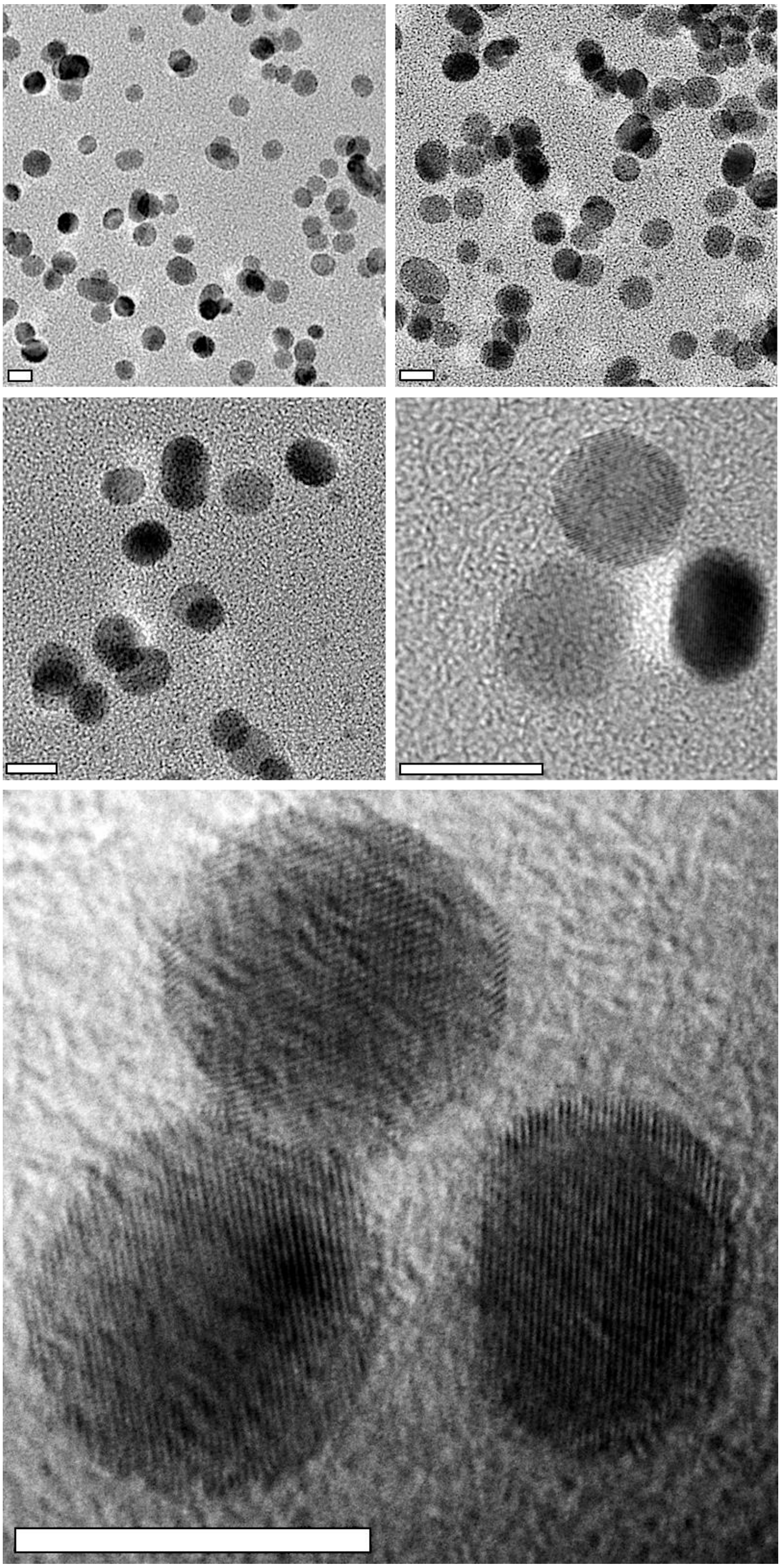

Figure S2. TEM images (different magnifications) taken from dispersed Pd nanoparticles in Teflon AF. Scale bars are 10 nm. Pd content ~ 8×10^{-3} vol. %.

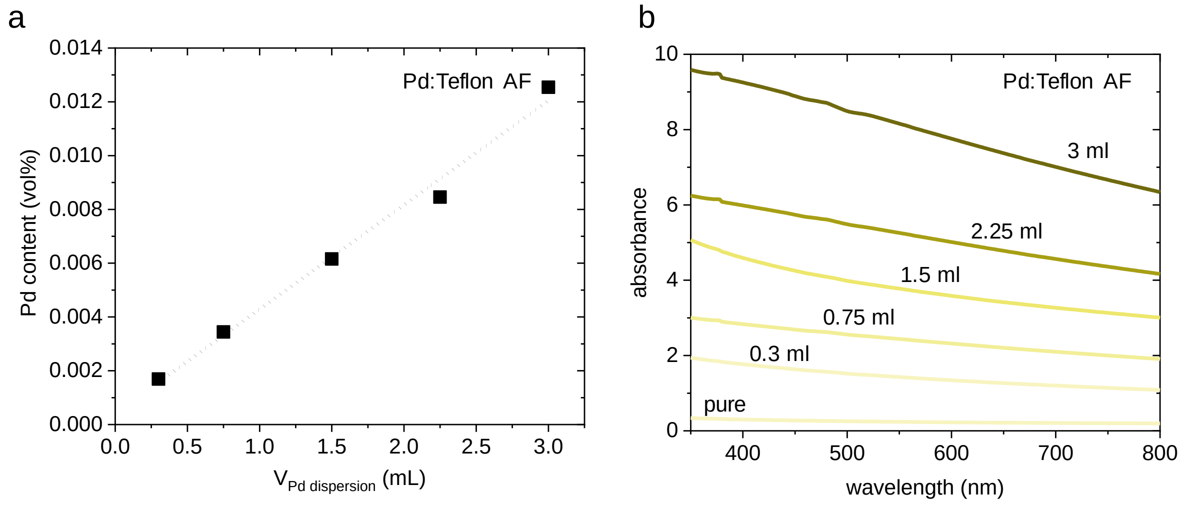

Figure S3. a) The Pd nanocube concentration in Pd:Teflon AF nanocomposites versus added volume of Pd nanocube suspension (isopropanol medium) after flow synthesis and medium exchange; b) UV-vis absorbance spectra of 100 μm thick Pd:Teflon AF nanocomposite plates with different added volume of Pd nanocube suspension (isopropanol medium).

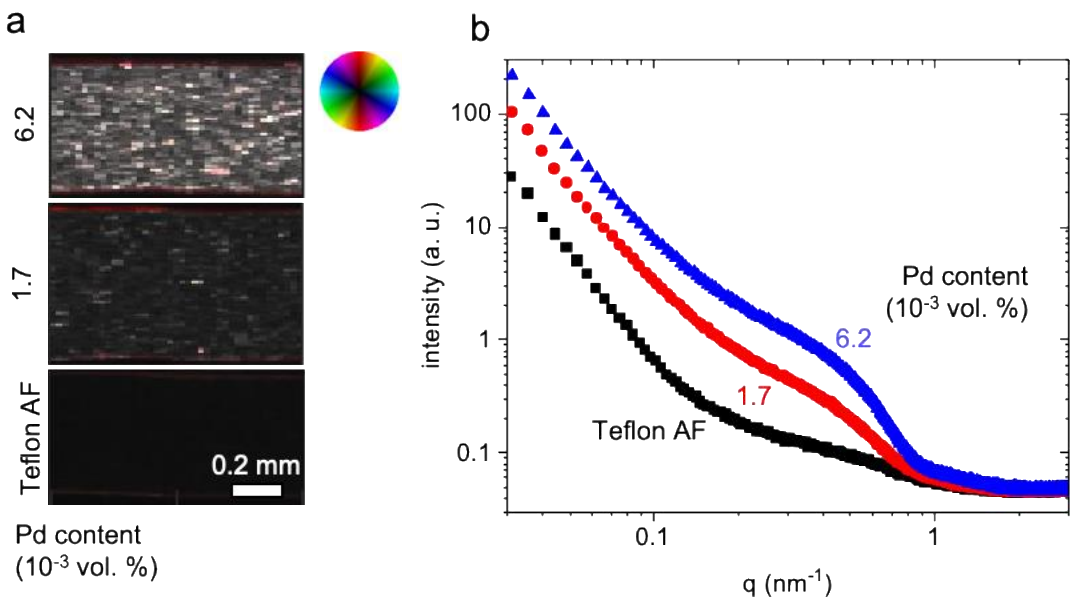

Figure S4. Small-angle X-ray scattering (SAXS) of Pd:Teflon AF nanocomposites. a) combined scanning-SAXS images of melt-pressed (combine the preferred orientation angle, the degree of orientation and the average scattering intensity in a hue-saturation-value representation according to the color wheel analyzed in a q-range of 0.2 to 0.69 nm^{-1}) and b) SAXS scattering curves of Pd:Teflon AF nanocomposites (raw data, i.e. before subtraction). Blue triangles correspond to a 6.2×10^{-3} vol % Pd particles and red circles to 1.7×10^{-3} vol % Pd particles.

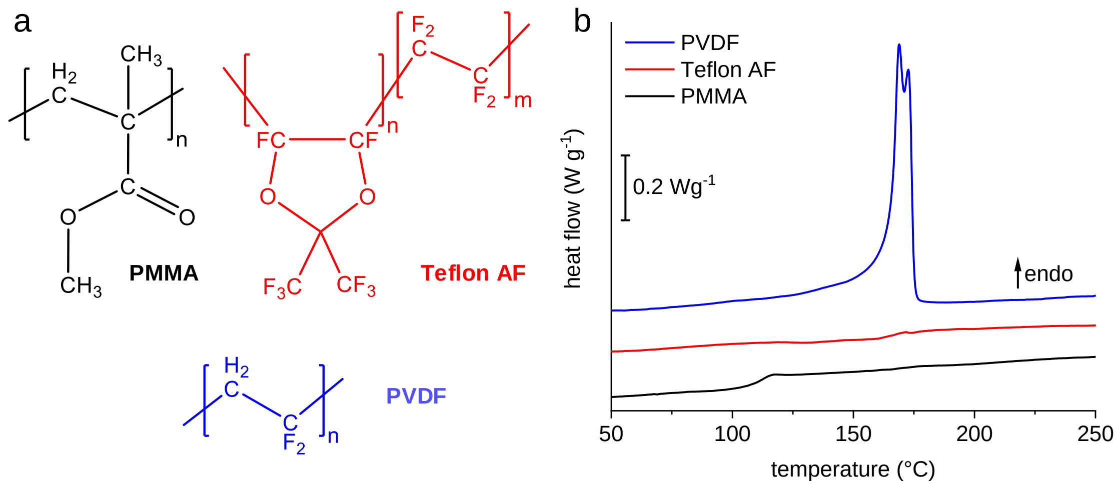

Figure S5. a) Chemical structure and b) heating DSC thermograms of PMMA, Teflon AF and PVDF.

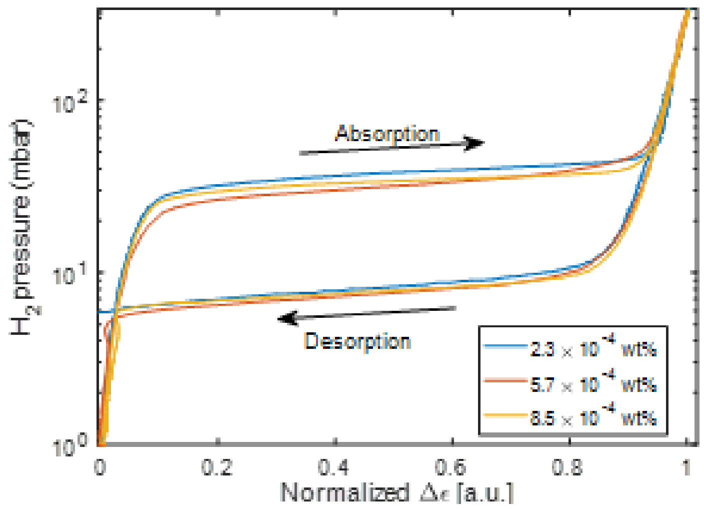

Figure S6. The pressure-composition isotherms of different Pd concentration in Teflon AF.

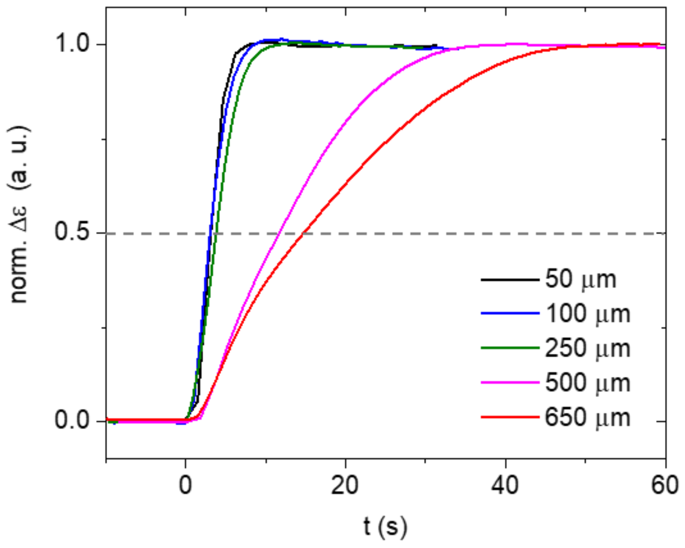

Figure S7. Normalized $\Delta\varepsilon$ of melt-pressed Pd:Teflon AF (3.4×10^{-3} vol. % Pd) plates upon a sudden increase in H_2 pressure from 0 to 100 mbar H_2 (the H_2 valve opens at t = 0).

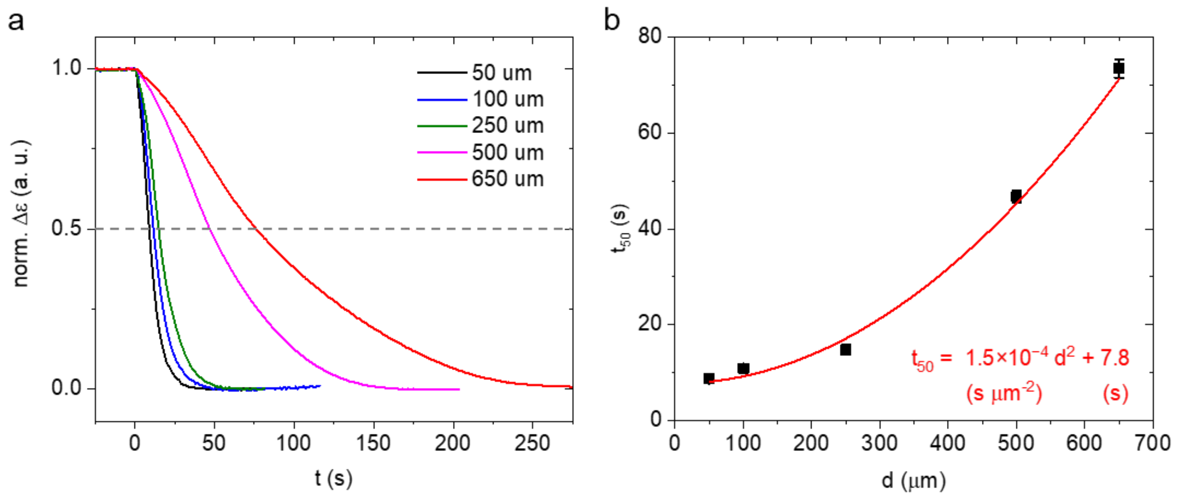

Figure S8. a) Normalized $\Delta\varepsilon$ of melt-pressed Pd:Teflon AF (3.4×10^{-3} vol. % Pd) plates during desorption; b) Corresponding sensor response time (H_2 desorption time) t_{50}.

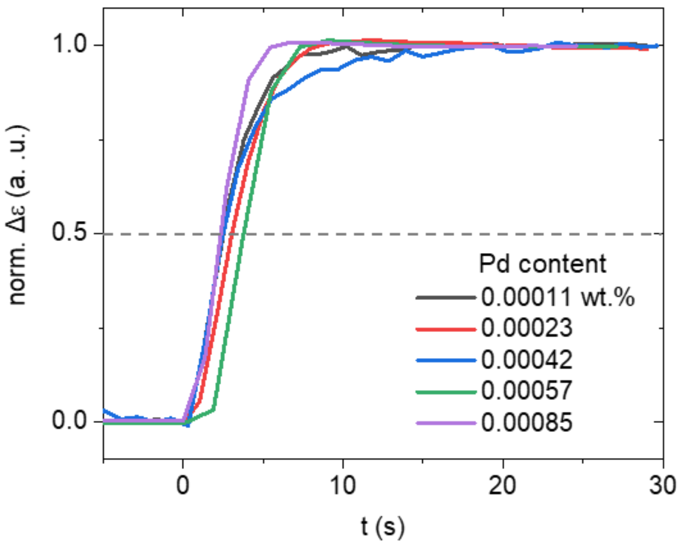

Figure S9. Normalized $\Delta\varepsilon$ of 100 μm thick melt-pressed Pd:Teflon AF plates with different Pd content.

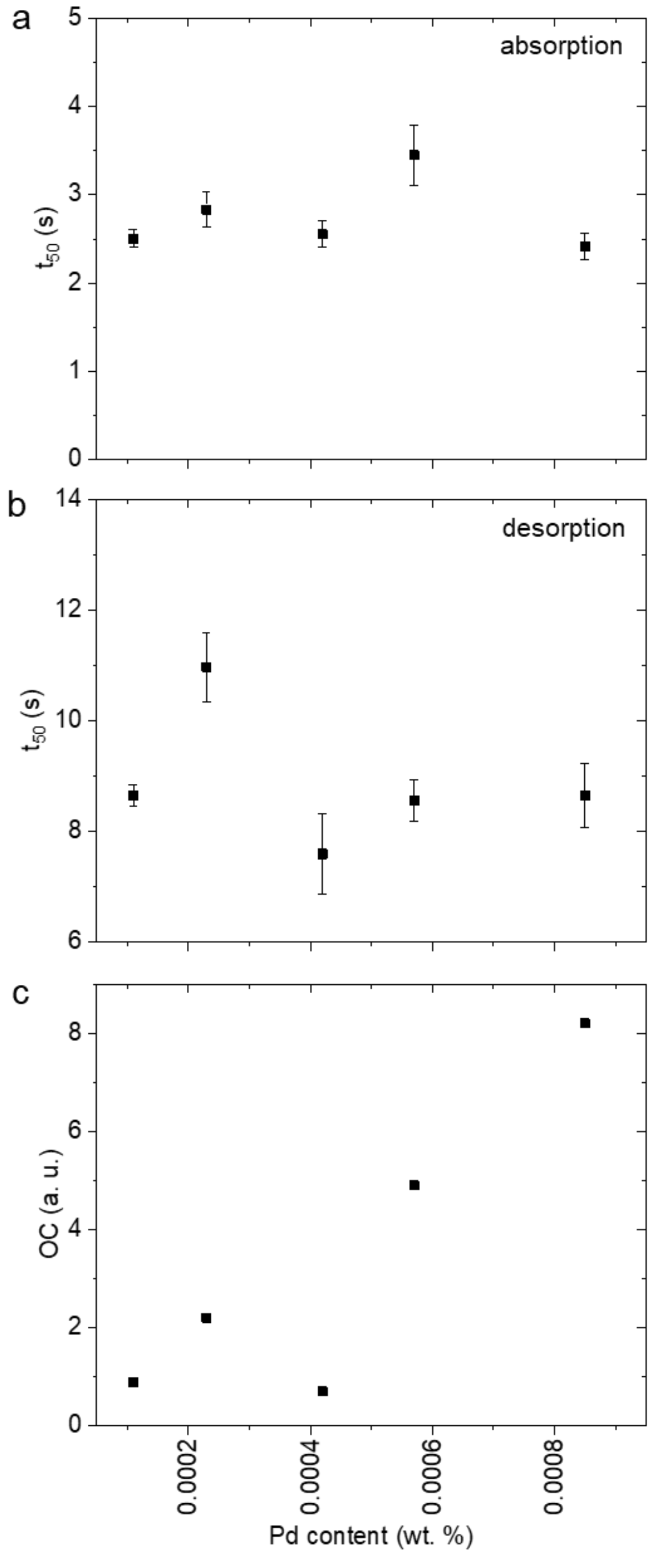

Figure S10. a) and b) H_2 absorption and desorption time (t_{50}), respectively and c) optical contrast of 100 μm thick melt-pressed Pd:Teflon AF plates with different Pd content.

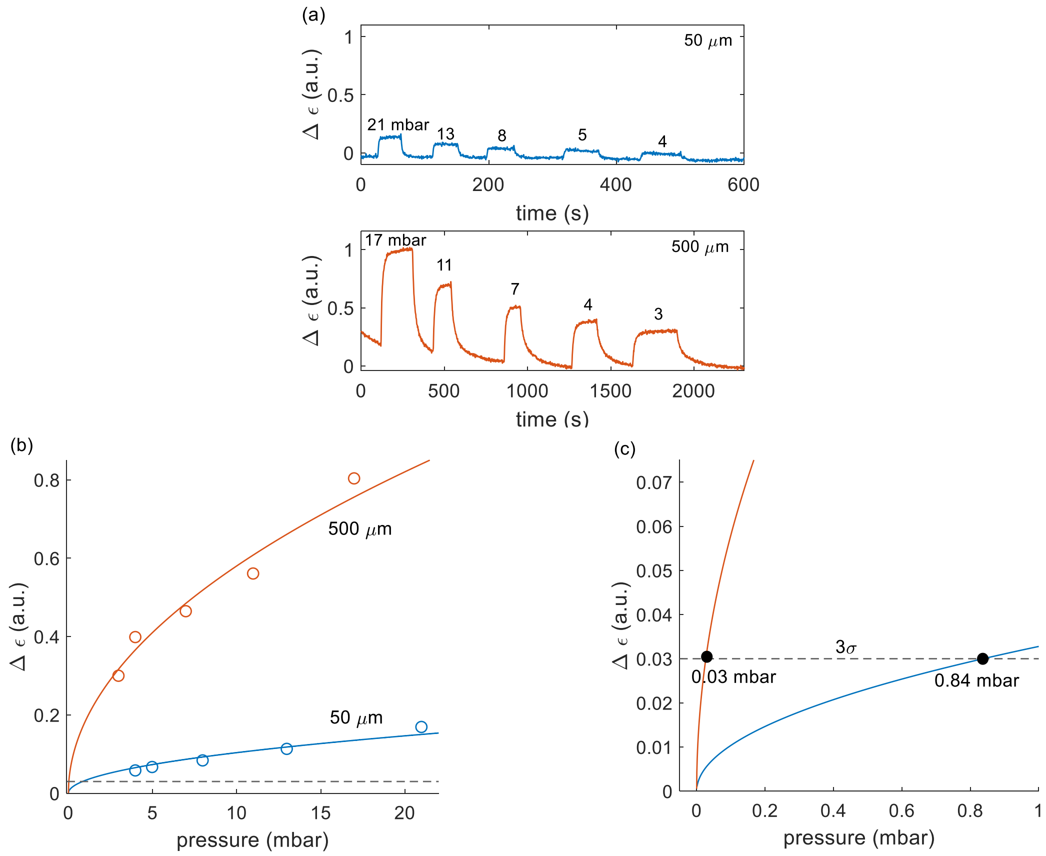

Figure S11. a) Sensor response of 50 and 500 um thick sensor at low hydrogen pressures, b) the optical responses *(Δϵ)* vs hydrogen pressure and c) a magnification of b) at very low pressure.

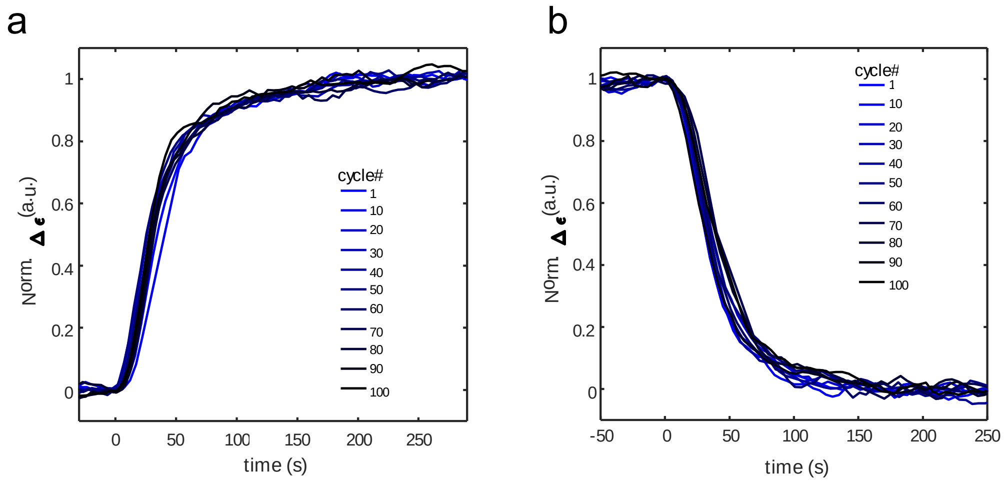

Figure S12. Normalized $\Delta\varepsilon$ of optical fiber cap Pd:Teflon AF (3.4×10^{-3} vol. % Pd) a) absorption and b) desorption at during cyclic exposure to 4 vol% hydrogen in synthetic air.
